# Evaluation of the national policy of single screening and treatment for the prevention of malaria in pregnancy in two districts in Eastern Indonesia: health provider perceptions

**DOI:** 10.1186/s12936-018-2426-y

**Published:** 2018-08-24

**Authors:** Jenny Hill, Chandra U. R. Landuwulang, Ansariadi, Jenna Hoyt, Faustina H. Burdam, Irene Bonsapia, Din Syafruddin, Jeanne R. Poespoprodjo, Feiko O. ter Kuile, Rukhsana Ahmed, Jayne Webster

**Affiliations:** 1 Department of Clinical Sciences, Liverpool School of Tropical Medicine, Liverpool, UK; 2 Eijkman Institute for Molecular Biology, Jakarta, Indonesia; 3 Department of Epidemiology, School of Public Health, Hasanuddin University, Makassar, Indonesia; 4 Mimika District Health Authority, Timika, Papua Indonesia; 5 Timika Malaria Research Program, Papuan Health and Community Development Foundation, Timika, Papua Indonesia; 6 Department of Child Health, Faculty of Medicine, University Gadjah Mada, Yogyakarta, Indonesia; 7 Disease Control Department, London School of Tropical Medicine and Hygiene, London, UK

**Keywords:** Malaria in pregnancy, Single screening and treatment, Acceptability, Health providers, Malaria prevention, Antimalarials, Dihydroartemisinin–piperaquine

## Abstract

**Background:**

Malaria in pregnancy has devastating consequences for both the expectant mother and baby. Annually, 88.2 (70%) of the 125.2 million pregnancies in malaria endemic regions occur in the Asia–Pacific region. The control of malaria in pregnancy in most of Asia relies on passive case detection and prevention with long-lasting insecticide-treated nets. Indonesia was the first country in the region to introduce, in 2012, malaria screening at pregnant women’s first antenatal care visit to reduce the burden of malaria in pregnancy. The study assessed health providers’ acceptability and perceptions on the feasibility of implementing the single screening and treatment (SST) strategy in the context of the national programme in two endemic provinces of Indonesia.

**Methods:**

Qualitative data were collected through in-depth interviews with 86 health providers working in provision of antenatal care (midwives, doctors, laboratory staff, pharmacists, and heads of drug stores), heads of health facilities and District Health Office staff in West Sumba and Mimika districts in East Nusa Tenggara and Papua provinces, respectively.

**Results:**

Health providers of all cadres were accepting of SST as a preventive strategy, showing a strong preference for microscopy over rapid diagnostic tests (RDTs) as the method of screening. Implementation of the policy was inconsistent in both sites, with least extensive implementation reported in West Sumba compared to Mimika. SST was predominantly implemented at health centre level using microscopy, whereas implementation at community health posts was said to occur in less than half the selected health facilities. Lack of availability of RDTs was cited as the major factor that prevented provision of SST at health posts, however as village midwives cannot prescribe medicines women who test positive are referred to health centres for anti-malarials. Few midwives had received formal training on SST or related topics.

**Conclusions:**

The study findings indicate that SST was an acceptable strategy among health providers, however implementation was inconsistent with variation across different localities within the same district, across levels of facility, and across different cadres within the same health facility. Implementation should be re-invigorated through reorientation and training of health providers, stable supplies of more sensitive RDTs, and improved data capture and reporting.

**Electronic supplementary material:**

The online version of this article (10.1186/s12936-018-2426-y) contains supplementary material, which is available to authorized users.

## Background

Malaria in pregnancy has adverse and often severe consequences for both mother and baby. An estimated 88.2 of 125.2 million pregnancies (70%) in malaria endemic regions occur in the Asia–Pacific region each year, of which 6.4 million pregnancies occur in Indonesia [1]. Indonesia has one of the most diverse malaria epidemiology’s globally. Of the 4.4 million pregnancies estimated to occur in areas with *Plasmodium falciparum* transmission nationally, 2 million are in areas with stable transmission and 2.4 million in areas with unstable transmission [1]. An estimated 6.3 million pregnancies occur in areas with *Plasmodium vivax* transmission and 4.3 million in areas in which *P. falciparum* and *P. vivax* coexist [1]. Both *P. falciparum* and *P. vivax* infections are associated with severe maternal anaemia, fetal loss and reduction in mean birth weight or low birth weight [2, 3]. Although the annual parasite incidence in Indonesia has fallen dramatically over the past 10 years, from 2.89 to 0.9 per 1000, and up to 72% of its population live in malaria free areas [4], the prevalence of malarial infection as determined by microscopy in some regions in Indonesia and Papua New Guinea is as high as 10–20% [5]. The harmful effects of malaria in pregnancy are preventable yet the Asia–Pacific region has no regional prevention strategy, with World Health Organization (WHO) recommendations relying on passive case detection and case management alongside the use of long-lasting insecticide-treated nets (LLINs) [6, 7].

Indonesia was the first country in the Asia region to have developed a national screening policy for prevention of malaria in pregnancy. First introduced in 2012, the policy consists of screening all pregnant women at first antenatal care (ANC) visit with either microscopy or a rapid diagnostic test (RDT) and treatment of parasite positive cases with the national recommended anti-malarial [8]. At the time of the study, quinine was recommended for treatment of malaria in the first trimester and dihydroartemisinin–piperaquine (DP) in the second and third trimesters [9]. The concept of the single screening and treatment (SST) policy is that the detection and treatment of malaria infections early in pregnancy will reduce the harmful effects to the growing fetus. On subsequent ANC visits, only women who have symptoms of suspected malaria, such as fever, are tested for malaria and positive cases are treated with either quinine or DP depending on the trimester of pregnancy.

An evaluation of the SST policy in Indonesia was undertaken to assess the acceptability and effectiveness of health providers in delivering SST to pregnant women through antenatal clinics in the routine health system setting. The findings from the qualitative study on acceptability and the perceptions of health providers on the feasibility of implementation of SST are presented here. The results from the quantitative study measuring effectiveness of antenatal clinics to deliver SST are published separately (Webster et al. [10]).

## Methods

### Study site and context

The study was conducted between February and July 2015 in two districts in two island provinces of Eastern Indonesia, West Sumba District in East Nusa Tenggara Province, and Mimika District in Papua Province. Papua Province has the highest burden of malaria in Indonesia, defined as moderate year-round transmission. Malaria transmission in West Sumba is low and focal or localized, and is the district with the lowest number of malaria cases in Sumba island [11, 12]. The study was nested in a clinical trial on alternative prevention strategies (Trial registration number ISRCTN 34010937) that was undertaken by the same research group in the same district in Papua and in an adjacent district in Sumba for convenience of logistics and availability of field staff (Ahmed et al. pers. comm.).

Mimika District lies in the south of Papua Province, covering 19,959 square kilometres consisting of highlands and lowlands. It has a rapidly growing population of an estimated 182,000 (2010 census [13]) Papuan and non-Papuan Indonesians, with the main occupations in commerce, business, retail and restaurants. The prevalence of parasitaemia among pregnant women at delivery is 16.8% (58% *P. falciparum* infections, 34% *P. vivax*, and 8% mixed infections), with 35% of these infections being associated with fever [3]. West Sumba District lies to the south of East Nusa Tenggara Province. The population of West Sumba is 121,901 (Statistics Indonesia 2015 [13]) and the main occupation is farming.

The main health facilities which deliver ANC services to pregnant women are hospitals, community health centres (known in Indonesian as *puskesmas*) covering about 30,000 people, sub health centres (*pustu*) which serve about 2–3 villages and 2000–3000 population, and health posts (*posyandus*) or community integrated services including antenatal care, which are held monthly or bi-monthly in villages. Mimika District has one District Government Hospital and one non-government general hospital, 23 health centres, 32 sub health centres and 129 health posts. West Sumba has 2 hospitals, 9 health centres, and 75 health posts. ANC attendance in Mimika and West Sumba is similar, with 84 and 87% of pregnant women making at least one ANC visit, respectively [14, 15]. Only a third of women make at least 4 ANC visits in Mimika; data for West Sumba was not available.

The SST policy applies to all pregnant women living in malaria endemic areas of Indonesia at their first antenatal care (ANC) visit, regardless of trimester, and treatment of parasite positive cases with the national recommended anti-malarial [8, 9]. At the time of conducting the study, quinine was recommended for treatment in the first trimester and is given as a 7-day regimen. DP (a co-formulated tablet containing 40 mg dihydroartemisinin and 320 mg piperaquine phosphate) is recommended for treatment in the second and third trimesters, and is given as a 3-day regimen with women receiving a dose of between 9–12 tablets/day based on body weight. All antenatal care services, including SST and passive case detection, are provided free to pregnant women attending public health facilities in malaria endemic regions in the country [16]. The policy states that SST should be delivered in any facility in malaria endemic areas that has an ANC programme (hospitals, health centres, sub health centres and health posts).

### Participants and study procedures

In-depth interviews (IDIs) were conducted with health providers working in the district hospital and three health centres in West Sumba and at seven health centres in Mimika. Health facilities were purposively selected to reflect the diversity of settings, including health facility size, urban rural location, geographic setting (highland and lowland), and number of malaria cases. Interviewees were purposively selected if they were involved directly or indirectly in the provision of ANC services, and included the following staff: the head of the health facility, doctors and midwives working in ANC (including the midwife coordinator), a laboratory technician, the malaria coordinator, a pharmacist, and in Mimika, the head of the drug store. Representatives of the District Health Office from each district were also interviewed.

Participants gave written informed consent prior to the interviews, starting with the head of the health facility and subsequently other staff in the same facility. One-to-one interviews with health providers were conducted in Indonesian using a semi-structured topic guide. Interviews aimed to assess health providers’: (1) acceptability of the SST strategy, (2) any adaptations to their working practices that were required to implement SST, and (3) any recommendations on factors to be considered to ensure more effective implementation. Interviews were digitally recorded and transcribed.

### Data analysis

The transcripts were entered into NVivo (QSR International) Version 11 for data management and analysis. Data from the IDIs were coded by two investigators (Hoyt and Hill) using a combination of pre-defined themes developed by two authors (Hill and Webster) based on the key research questions. Any differences in coding were discussed until consensus was reached.

The feasibility framework developed by Bowen et al. [17], describing eight areas to be considered when conducting feasibility studies, was adapted and used to structure the analysis. The following seven areas were selected: acceptability, demand, implementation, practicality, integration, adaptation, and expansion or scale-up. The definitions of each area and key outcomes are provided in Table 1. Additional themes that emerged from the data were developed using content analysis [18]. During data analysis, all study participants were assigned an anonymous code, and quotes are identified by health provider role and district.

**Table 1:** Feasibility framework areas and outcomes of interest. Source: Adapted from Bowen et al. [5]

Area	Description	Sample outcomes of interest	IDIs
Acceptability	To what extent is a new idea, programme, process or measure judged as suitable, satisfying, or attractive to programme deliverers? And perceived as such to users by deliverers?	Satisfaction Intent to continue use Perceived appropriateness	+++
Demand	To what extent is a new idea, programme process, or measure likely to be used (how much demand is likely to exist)?	Fit within organizational culture Perceived positive/negative effects on organization Actual use or intention to use Perceived demand	++
Implementation	To what extent can a new idea, programme, process, or measure be successfully delivered to intended participants in some defined, but not fully controlled context?	Degree of execution Success or failure of execution Amount/type of resources needed to implement	±
Practicality	To what extent can a new idea, programme, process, or measure be carried out with intended participants using existing means, resources, and circumstances without outside intervention?	Factors affecting ease or difficulty of implementation Efficiency, speed or quality of implementation Positive/negative effects on target populations Ability to carry out interventions Cost analysis	+
Adaptation	To what extent can a new idea, programme, process, or measure be or be likely to be implemented as designed?	Degree to which similar outcomes obtained in a new format Process outcomes comparison between interventions in two populations	++
Integration	To what extent can a new idea, programme, process, or measure be integrated within an existing system?	Perceived fit with infrastructure Perceived sustainability	++
Expansion (scale-up)	To what extent can a new idea, programme, process, or measure be expanded to provide a scaled-up programme?	Cost to organization and policy bodies Fit with organizational goals and culture Positive/negative effects on organization Disruption due to expansion component	+

Key: scale for contribution of data through IDIs: ±, IDIs contribute limited data (low); +++, IDIs good source of data (high)

## Results

In-depth interviews were conducted with 86 health providers—25 in West Sumba and 61 in Mimika (see Table 2). Key themes emerging from the analysis are described here by each area, as defined in the feasibility framework: Acceptability and demand (Table 3), implementation and practicality (Table 4), and adaptation, integration and scale-up (Table 5). Quotes supporting each of the themes and sub-themes are provided in Additional file 1.

**Table 2:** Interviewees by cadre in West Sumba and Mimika

	West Sumba	Mimika	Total
Head of DHO	1	1	2
Head of health facility^b^	0	7	7
Doctor	3	7	10
Lab technician	4	7	11
Malaria coordinator	4	8	12
Midwife coordinator	5	8	13
Village midwife	3	7	10
Pharmacist	5	8	13
Head of drugstore^a^	0	8	8
Total	25	61	86

^a^In West Sumba, the pharmacists were also in charge of the drug stores

^b^In West Sumba, Heads of health facilities were excluded as they were not medically qualified

**Table 3:** Summary of key findings: acceptability and demand

Area	Themes	Sub-themes
*Acceptability*		*To what extent do health providers accept SST?*
	Of SST for control of malaria in pregnancy	SST is good because it is important to detect malaria early in pregnancy, pregnancy is risky time; SST should be continued; SST is a good policy but only if it is in fact being carried out and the quality is improved
	Of RDTs vs microscopy	RDTs are not always accurate; RDTs are easy to use and a good alternative if there are no lab services or for use in the field; RDTs are useful but we may still need to confirm result with microscope
	Of DP	Most participants reported no challenges with DP use; DP was not always immediately effective DP can produce mild side effects in some women (nausea, vomiting, dizziness)
*Demand*		*To what extent is SST perceived to have positive effects, to be used, and demanded by PW?*
	For SST by pregnant women (perceived by health providers)	Awareness about screening is low and some pregnant women don’t go for screening despite being advised, e.g. transportation costs; lack of motivation to attend health facilities; women question why they are being tested when they have no complaints

**Table 4:** Summary of key findings: implementation and practicality

Area	Themes	Sub-themes
*Implementation*		*To what extent is SST being carried out in health facilities, and where?*
	SST	Pregnant women are being screened for malaria on their 1st ANC visit (regardless of symptoms); after the 1st visit, they will only be screened for malaria if they present with symptoms; SST is reportedly not being implemented at all or not consistently
	SST at health posts/village level	Pregnant women are not being screened at health posts; only carried out at health posts if RDTs are available; pregnant women are told to go to health centres for screening; screening is being done at health posts using RDTs; in some village settings, only symptomatic women are being screened; challenges with implementation at village level: a) Limited RDT stocks or complete stock-outs; b) Lack of staff (trained staff, lab technicians)
	RDT availability	RDTs have never been used; are not available/current stock outs for RDTs and previous stock outs across facilities/areas; RDTs expired before being used or facilities receiving RDTs close to expiry
	Anti-malarial prescription at different facility levels	Anti-malarials not available at health posts; pregnant women must to go to health centres to receive treatment; treatment prescribed by a doctor; doctors and/or midwives provide pregnant women with prescription (at ANC); women collect anti-malarials from pharmacy; anti-malarials cannot be prescribed without confirmation from a diagnostic test for malaria
*Practicality*		*To what extent is SST being carried out using existing resources?*
	RDT vs microscopy	Microscopy is main method of screening at health centres, or is used primarily but sometimes RDTs are used when the electricity is out or lab services are not available; when available RDTs are used for screening at health posts/sub health centres; RDTs are often administered by midwives, or lab technician (reported at one location); RDTs are not being used at health posts
	SST at different levels of health facility	SST should be done at health posts as they are more accessible; SST at both health posts and health centres; at health centres as they have microscopes and staff; SST at all facilities is good if you have the resources; SST at home would be the best option
	DP for treatment	DP is prescribed for treatment in 2nd and 3rd trimesters; DP stocks were mostly stable, but a few participants reported occasional stock outs; DP is well tolerated and effective; health provider concerns about completing doses of anti-malarials; DP has shorter dosing regimen than quinine, could be better for compliance

**Table 5:** Summary of key findings: adaptation, integration and scale-up

Area	Themes	Sub-themes
*Adaptation*		*To what extent have changes been made to existing systems to implement SST?*
	Replacement of microscopes with RDTs	Can microscopes be replaced with more sensitive RDTs?
	Anti-malarials given by midwives in villages	Can midwives deliver drugs at the village sites?
*Integration*		*To what extent has SST been integrated into the existing system?*
	SST at health centres (current strategy)	ANC and labs work together to screen pregnant women; midwives carry out screening and malaria coordinator is responsible for reporting
	Anti-malarials being given by midwives	Not the role of midwives to prescribe/distribute anti-malarials; important for medications to pass through pharmacy; if no doctor is available, then a midwife can prescribe medications but doctor should be consulted; midwives can prescribe anti-malarials to pregnant women
	SST at village sites	Midwives request drugs and RDTs from health centre pharmacy/drugstore to take to health post/sub health centres
	Quantification of supplies	RDTs/anti-malarial orders based on the monthly consumption reports by ANC; orders placed by pharmacy to DHO quarterly; RDTs used for all patients/not exclusively pregnant women
	SST indicators into HMIS	Indicators collected by ANC at health centres and health posts No. ANC attendees No. received SST by ANC visit no SST recorded in ANC booklet and/or cohort book Diagnostic technique (RDT or microscope) recorded at some facilities but not others Indicators collected at village sites No. ANC attendees, No. received screening, positive/negative results Indicators collected by the lab Lab does not specifically collect data on PW, it reports general malaria data Health facility reporting ANC collects data on SST, malaria coordinator compiles the report but collates all malaria data (no specific report that links SST with pregnant women or ANC visit)
*Expansion (scale-up)*		*To what extent has or can SST be scaled up and expanded?*
	Regular RDT supplies	Can RDTs be supplied regularly and stock-outs avoided?
	Health provider roles	Malaria coordinator is primarily involved in reporting on malaria in general; often has another role such as lab tech or nurse; sometimes directly involved with SST program Doctors are involved with the treatment of malaria in pregnancy Midwife coordinators carry out screening and collect data from various sites; village midwives’ roles vary depending on what malaria related activities are done at the village sites
	Health provider training	Never been trained on malaria screening/use of RDTs; received training on malaria screening; not received formal training but learned from colleagues
	HMIS	Can the HMIS system be improved to reduce stock outs of RDTs and monitor SST program?
	Sustainability of funding	Global Fund may have stopped funding SST related logistics; no specific budget for malaria in pregnancy

### Acceptability: to what extent do health providers accept SST?

Most health providers thought SST was a beneficial policy, highlighting the importance of detecting and treating malaria infections during pregnancy, which was regarded as a risky period. In Mimika, one midwife described the value of screening for malaria in an area where infections were often asymptomatic.

*"R: Yes, I think malaria screening is the best method for malaria prevention in pregnancy. I: Why? R: It is because in Timika people already have immunity for malaria so although the patient has no symptom but actually the patient has parasite inside her/his body…For example, the patient has no symptom but the screening result is malaria mixed [infection]. So, I think screening is very important.” Village midwife, Mimika.*

Some providers thought SST was a good policy but only if it was carried out comprehensively, stating that the quality needs to be improved. Health providers not involved in providing ANC services were less familiar with the SST strategy and/or concept. For example, a pharmacist in Mimika said that only symptomatic pregnant women should be screened for malaria.

The method of screening was an important consideration in the acceptability of the SST strategy. The type of RDT used by the national programme mostly involved a combination HRP2/pLDH based RDT, in which there is separate result lines for *P. falciparum* and for other Plasmodium species, which are predominantly due to *P. vivax* in these areas. RDTs were widely considered inferior to microscopy as they are unable to detect low parasite densities and some parasite species, causing doubt about the accuracy of results and the need for microscopic confirmation. Laboratory technicians, malaria coordinators and doctors generally regarded RDTs as a last resort, if no other appropriate tools were available.

*“I: In your opinion, is RDT used for diagnosis as an alternative to slides? R: If the result of RDT is not significant, we would confirm again by using microscope. I: Can RDT be used as an alternative without using slide again? R: I think, it can be, since RDT is the recommended tool. If the result is doubtful, we have to ensure it by using slide. However, if the line of RDT shows on vivax or falciparum clearly, we can use it as the result of diagnosis.” Malaria coordinator, Sumba.*

*“R: ..Screening test by using RDT kit… if there is no other appropriate tool to perform the screening test then using RDT kit is our last option to take. From the effectiveness and specification, RDT is not as good as DDR [microscope]. DDR has become our gold standard…. In fact, if the screening test aided by RDT kit, we still need to reconfirm the result by using DDR [microscope] in the last process. If we found some positive test result we should reexamine the blood slide to see if it’s true, like a confirmation.” Doctor, Mimika.*

Midwives were more pragmatic, making a distinction between screening for SST and testing of symptomatic cases.

*"I: What do you think about the RDT examination? Is RDT allowed to be used at posyandu [health post]? R: In my opinion, RDT is only for screening. I agree if RDT is only used for screening because ideally for malaria examination we have to use microscope not RDT because the result is not really accurate. That’s why we have to look at the clinical signs when we give the treatment.” Midwife coordinator, Mimika.*

Midwives and a few laboratory technicians acknowledged that RDTs were a good alternative to microscopy in some settings, e.g. facilities with no laboratories, during power cuts and at village level (sub health centres and health posts). RDTs were thought to be simple to use, time saving and transportable and, if made available at village level, would avoid the need to refer symptomatic women in remote areas without laboratory services, which delayed treatment.

Most providers reported no major challenges with using DP for the treatment of malaria in the second and third trimester, although there were reports of DP producing mild side effects in some women (nausea, vomiting and dizziness). In comparison to quinine, the side effects were reportedly less severe (‘better than Kina’) [quinine]. Providers said pregnant women preferred DP to quinine as the side effects lasted for fewer days given its shorter regimen. A malaria coordinator in West Sumba thought nausea reduced adherence to the full regimen, and midwives in Mimika advised women to eat and drink sugared water to help manage the nausea. One participant reported that DP had failed on several occasions, saying that it did not always work immediately and that women remained infected (microscope positive) for up to 4 days before the effects were seen.

### Demand: to what extent is SST perceived to have positive effects, to be used, and demanded by pregnant women?

Health providers were asked about the uptake of SST among pregnant women. While some providers thought pregnant women were willing to come for screening, most reported low awareness of SST and a lack of motivation for women to attend health facilities unless they were sick. While many ANC services were provided at village level, SST was not routinely provided so women were counselled on the importance of SST and referred to a health facility, yet women often didn’t go due to transport costs or other reasons. Providers felt that, despite receiving counselling, pregnant women did not understand the need for testing when they had no symptoms, and required more education.

*“R: Besides screening, we also have communication and counseling with the pregnant women, because they are unwilling to come to puskesmas [health centres], unless when they are sick. So, they are recommended to come here so that they understand about the danger of malaria, even it could cause miscarriage. That’s all we do, because we cannot change their lifestyle in an instant.” Malaria coordinator, West Sumba.*

### Implementation: to what extent is SST being carried out in health facilities, and where?

#### SST implementation by facility level

Implementation of SST in both sites has been inconsistent, with some providers in West Sumba expressing doubt about how extensively the policy was being implemented and/or about the quality of implementation.

*“I: Is the malaria screening test on pregnant women without symptoms has been the best choice according to your opinion? R: … It’s good when you actually do it, but whether it’s really being done is unknown. I think this is a good policy, but the quality needs to be increased and also the power behind it. The workers also need to be trained.” DHO, West Sumba.*

At the time of the study, several facility staff said they were not implementing SST, including staff at the regional hospital in West Sumba. However, there were inconsistencies in reports between some health providers at the same facility. Generally, providers not directly involved with antenatal care had less knowledge regarding the implementation of SST at their facility or related health posts whereas midwives were a more reliable source regarding whether SST was being implemented or not as they were directly involved. Understanding of the SST policy at health facilities was generally high among midwife coordinators and village midwives in both sites.

SST was almost exclusively being implemented at the health centre (*puskesmas*) level, predominantly using microscopes for screening. According to midwives in Mimika, screening was only carried out at village level if RDTs were available, adding that while RDTs had previously been available they were no longer in stock.

*“I: Do you think SST is performed in this puskesmas [health centre]? R: It is… when the recipient go to the puskesmas, even if there is no RDT kit available, there is laboratory technician who can perform the examination using microscope. So SST is running in puskesmas, but in posyandu [village post] it is limited to the availability of RDT.” Midwife coordinator, Mimika.*

Only one health facility in Mimika reported that SST was consistently implemented at village health posts (*posyandu*).

#### RDT availability

Lack of RDT supplies was cited as a major limitation to provision of SST in health posts, with universal reports that RDTs had either never been supplied or were ‘rarely’ available. On the day of the interview, availability of RDTs was reported in only three of ten health facilities (excluding the hospital). When RDTs had been provided in the past, several facilities reported having received RDT stock close to expiry. RDTs were not used exclusively for screening pregnant women, they were used for any symptomatic patients at the village posts and at the health centres if the lab service was not available, e.g. if no electricity.

*“I: So how did you count the RDT for pregnancy at the Lab? R: … for example, I have to supply each pustu [sub health centre] with 100 sticks of RDT kit, of that 100 sticks pregnant patients are in it. I: 100 pregnant women to be examined? R: not 100 pregnant women, but of that 100 sticks it could be for general patients, it could be for pregnant women. And if it’s on shortage we may request it. I: Oh. So it’s not particularly meant for the pregnant women. R: No. I: Just in general? R: In general.” Malaria coordinator, Mimika.*

*“I: So, …the 50 RDTs are not for pregnant women only, but for general patients also? R: It’s for general, including pregnant women who come with clinical symptoms. But most of them would be referred to puskesmas [health centres] to be checked, and it would be good for recording and reporting. It’s more about optimum services as well.” Pharmacist, West Sumba.*

#### Malaria treatment by facility level and cadre

All providers stated that anti-malarials could not be prescribed without confirmation of malaria with a diagnostic test, whether performed at a health centre or at a village health post. At health centres, prescriptions were provided by doctors and/or midwives, and women then collected the drugs from the pharmacy. Women who tested positive at health posts were referred to a health centre for treatment, since village midwives do not carry curative medicines, only paracetamol, iron and vitamins. The exception was if a doctor was present at the health post then anti-malarials could be given in the village, but this was reported by only one respondent in Mimika and was not common practice.

*“I: Well, what if the midwives do some screening test and then they found that the patients are positively infected. Afterwards, the drugs are given immediately. What do you think about it? R: The midwives do not have in their possession the malarial drugs. The drugs are only stocked in Pharmacy and never be directly given to them, unless they are on field. They need to be there with the doctors, otherwise the malarial drugs cannot be issued. We only give them RDT kit so that they can use it to diagnose the patients. And you know, the positively infected ones will be referred to puskesmas [health centre]. If the midwives pay visit to posyandu [health post] along with the doctors, then the malarial drugs can be issued.” Pharmacist, Mimika*

### Practicality: to what extent is SST being carried out using existing resources?

#### Use of RDTs and/or microscopy for SST

Microscopy was the main method of screening pregnant women at health centres, since microscopes and reagents were made available for passive case detection. When available, RDTs were said to be used for screening pregnant women at sub health centres and health posts, though in some areas participants reported that screening was not being done at health posts.

*“I: why did you not take RDT kits with you to posyandu [health post]? R: that’s because*—*I don’t know, we were always told that RDT stock is limited, and because malaria is diagnosed using microscope at the laboratory instead of using RDT. I mean RDT is less accurate, more microscopical examinations are using microscope. There’s no way we would take microscope to pustu [sub health centre], to remote areas. Until this moment there are still no RDT available… I: So, since you started working in this puskesmas [health centre], you never brought RDT kit to posyandu [health post]. R: Never.” Village midwife, Mimika.*

Even where RDTs were being used in the villages, lack of confidence in test results meant midwives would refer suspected cases to a health centre for microscopy. Both sites reported that RDTs were used at health posts for symptomatic malaria and not specifically for screening pregnant women.

*“R: RDT kit is used here and pustu [sub health centre], every patient who is infected by malaria is diagnosed with RDT kit, whether the pregnant women or the common patients.” Malaria coordinator, Mimika.*

#### Use of DP for treatment in pregnancy

DP was variably referred to as ‘DP, ‘DHP’, ‘Darplex’, ‘OAM’, or ‘the blue drug’. All cadres demonstrated good knowledge of the dosing regimen by body weight and that use was restricted to the second and third trimesters. There were several reports of stock outs of DP, when quinine had to be used in place of DP. DP was said to be well tolerated and widely regarded to be effective in both sites.

#### SST at different levels of the health system

There were diverse views on which level(s) of health facility should provide SST, ranging from health posts being closest to homes, to a combination of health posts and health centres, to health centres and sub health centres only. Arguments for health centres were that services were always available and they have the resources (microscopes and staff), whereas health posts were conducted for only a few hours at a time therefore accessibility was limited. Midwives at two health centres in Mimika said SST was only provided at the health centres and sub health centres, since ANC was not provided at the health posts attached to those facilities. Ultimately the delivery of SST below the level of health facilities equipped with microscopes would be reliant on a stable supply of RDTs.

### Adaptation: to what extent have changes been made to existing systems to implement SST?

To some extent relatively few changes were made to existing systems to implement SST, with the least changes occurring at the higher levels of the health system, i.e. hospitals and health centres, where existing lab and prescribing practices have been utilized. Where SST was being implemented at sub health centres and/or health posts, the key adaptation has been to the role of midwives who perform the RDTs. However, many midwives in both sites had no formal training on either malaria in pregnancy, SST or the use of RDTs with some saying they learned how to use RDTs from colleagues. Midwives generally do not prescribe anti-malarials or other curative drugs, this was still being done by doctors.

### Integration: to what extent has SST been integrated into the existing system?

#### Health provider roles in SST delivery

None of the health providers interviewed thought that RDTs could replace microscopy for screening pregnant women given their lack of sensitivity and inability to detect density of infections and identify some species. However most conceded that where and when RDTs were available they could be used by village midwives for screening pregnant women at subhealth centres or health posts. As pregnant women who tested positive were currently referred to a health centre to collect prescription drugs, full integration at village level would require village midwives to be given the mandate and training to prescribe anti-malarials.

#### Quantification, procurement and supply of RDTs and DP

Procurement and distribution of RDTs and anti-malarials was said to be managed by the pharmacist and/or head of the drug store. RDTs and anti-malarials were ordered based on the monthly consumption reports provided by health facility ANC clinics and orders placed by the pharmacy (or head of drug store) to the District Health Office on a monthly or quarterly basis (usually quarterly). Midwives submitted monthly reports and requests for RDTs and anti-malarials from the health centre pharmacy or drug store to take to sub health centres and health posts. Both the District Health Office and facility heads claimed there was no specific budget for malaria in pregnancy, any requirements were integrated into the Maternal and Child Health programme budget.

#### Data collection and reporting on SST

Responsibility for reporting to the District Health Office on the implementation of SST lies with the malaria coordinators, alongside reporting on insecticide-treated net distribution and malaria cases. It seems existing health providers were nominated for the role as malaria coordinator, therefore their knowledge and depth of this role varied widely.

Midwives described recording ANC services provided to each pregnant woman, including SST at first ANC visit as well as screening of suspected malaria cases, and compiling the data into a report given to the malaria coordinators.

*“I: Does Kesga (Register Kesehatan Ibu dan Anak) [ANC register] gain the report about pregnant women who come at 1st and be screened? R: Yes. The data here are about malaria and HB testing. Whether the result is vivax positive, falciparum positive, or mix, that’s it. I: Could we see in the data for the pregnant women who come at 1st and be tested? R: The data about all pregnant women have been tested. Not only for pregnant women who visit at 1st [visit].” Midwife coordinator, Sumba.*

The diagnostic method (RDT or microscopy) was recorded at some health facilities but not in others. At health posts, a few providers said they recorded the number of pregnant women who received screening and the test results, but not always the ANC visit number. Laboratories on the other hand did not record pregnancy status only general malaria data. The malaria coordinator was responsible for collating all malaria data so that there was no specific report on malaria in pregnancy, and SST data were not included in the health management information system (HMIS) and general malaria reports generated at health facility or district level.

### Expansion: to what extent can SST be scaled up and expanded?

Several key themes emerged from the interviews that are relevant to the expansion of SST beyond current levels of implementation: review of village midwife roles particularly with respect to administering anti-malarials, reorientation and training; RDT sensitivity and availability; improved data capture and reporting of SST and integration of relevant indicators into the national HMIS; and sustainability of funding. These themes are explored further in the discussion.

## Discussion

The implementation of SST as reported by health providers in both sites was inconsistent, with least extensive implementation in West Sumba compared to Mimika. There was apparent wide variation across different geographic localities within the same district, at different levels of health facilities, and variation in reports from different health provider cadres within the same health facility, with midwives providing the most consistent reports of how, where and why SST was or was not being implemented. These findings are consistent with the quantitative evaluation of SST conducted in the same sites (Webster et al. [10]). The study provides important information for the national malaria and reproductive health programmes in Indonesia to strengthen delivery of the SST strategy, together with lessons learned to inform the roll-out of any new malaria in pregnancy interventions in the future (Hoyt et al. pers. comm.).

While health providers across all cadres found the SST strategy to be acceptable, West Sumba and Mimika have very different malaria epidemiology’s which may contribute to differences in the perceived benefits of SST for individual pregnant women and for achieving a reduction in the overall burden of malaria in pregnancy. A study conducted in southwest Sumba prior to the start of the clinical trial found malaria prevalence was 3.0% by RDTs and field microscopy, 5.0% by expert microscopy and 7.0% by nested PCR [19]. However, preliminary analysis of data from a subsequent trial found that malaria prevalence detected by RDTs in South West Sumba was lower than previous findings (< 1%) (Ahmed, unpublished data). It is possible that similar falls in slide positivity rates were experienced at health facilities in West Sumba and affected overall perceptions of risk among health providers. Mimika on the other hand has higher levels of transmission [3], such that health providers may perceive the pregnant population to be at higher risk in this setting.

At the time of the study, SST was predominantly being implemented at health centre level in both districts, where laboratories were equipped with microscopes for the general population, however it was not provided at the regional hospital in South West Sumba. Implementation at sub health centre and health post levels was limited; SST was reportedly provided by village health posts attached to less than half the health facilities in Mimika and in only one health facility in West Sumba. These reports are consistent with findings from the observational studies in ANC clinics in the same districts, which showed poor implementation in health posts in both sites, with less than 10% of first ANC visits screened, and no screening at the hospital in Sumba (Webster et al. [10]). In rural settings like South West Sumba, where most pregnant women attend health posts for ANC, this represents a missed opportunity to provide SST to most women.

Lack of availability of RDTs was cited as the main factor preventing provision of SST at health posts, with staff at only two of the 11 health facilities reporting any RDTs in stock on the day of the interviews. Again this finding is consistent with the quantitative study which showed that most screening conducted at first ANC visit in both sites was done by microscopy (Webster et al. [10]). The proportion of first ANC visits screened by an RDT vs microscopy were 1.1 and 1.2% in Mimika and West Sumba, respectively, and all were performed at health posts. Notwithstanding the unavailability of RDTs, all health provider cadres expressed a lack of confidence in RDT test results such that midwives would refer symptomatic patients with a negative RDT result to a health centre for confirmation by microscopy. Poor acceptability and adherence to RDT test results has also been reported in similar studies in pregnant women in Kenya and Malawi [20, 21]. Ultra-sensitive diagnostic tests that have recently become available on the market (e.g. Alere™ Malaria Ag Pf) and may increase the confidence in RDT results by health providers, although similar tests for non-falciparum species are still under development. Furthermore, the implementation of any malaria screening strategy in Indonesia will require a change in organizational culture and attitudes to malaria RDTs if to succeed.

A major challenge in the roll out of the SST strategy appears to be insufficient training of all frontline health providers, with many midwives in both sites reporting no formal training on either general malaria in pregnancy, nor SST or the use of RDTs, with some saying they learned how to use RDTs from colleagues. A further consideration for the effective delivery of SST at village level is the role of midwives in the provision of anti-malarials. Currently, village midwives are not mandated to prescribe anti-malarials so unless a doctor is available during the bimonthly health posts which deliver community integrated services including ANC, pregnant women who test positive are referred to a health centre to collect anti-malarials. However, there is no guarantee that women will make that referral visit meaning this practice leaves some women untreated.

Obtaining an accurate assessment of the extent of SST implementation in Indonesia is hampered by the lack of adequate indicators, tools and reporting of SST within the national HMIS. There is also a disconnect in data capture between antenatal care and malaria programmes. While ANC data compiled by midwives includes SST by ANC visit number and test result, these data are not captured nor reported in the malaria reports prepared by the malaria coordinators. Similarly, laboratories do not record pregnancy status in the lab registers used to record malaria test results. The uncertainty expressed by District Health staff in West Sumba about whether SST was being implemented at village health posts within the district highlights the lack of coverage data. The use of malaria screening data at the first antenatal visit may be a potentially useful programmatic tool to track malaria transmission intensity in the region [22].

Health providers perceived a low demand for SST among pregnant women, citing that they did not understand the need to have a test if they had no symptoms. Village midwives who referred women to health centres to be tested were doubtful women would go as instructed, and this contributed to the belief among many providers that SST should be provided at village level, some going as far as to say SST should be provided in the home. These findings suggest the need to raise awareness among women of child bearing age of the importance of screening, and of attending ANC even if not sick. Health provider reports of DP causing mild side effects in some women (nausea, vomiting and dizziness) will influence adherence in pregnant women and limit the effectiveness of this strategy. Problems with side effects to sulfadoxine–pyrimethamine used for intermittent preventive treatment in pregnant women was a major barrier to its uptake in sub-Saharan Africa [23]. The advice given to women by midwives in Mimika to eat or drink to manage nausea should be used to inform messaging around SST. Reasons for the reports of ‘failure of DP’, where women remained infected (microscope positive) for up to 4 days before the effects were seen, were not clear and may relate to quality of the drug, storage conditions and perceptions among health providers of the efficacy of DP, which will need to be addressed.

To improve the effectiveness of the SST strategy, priority areas include clarification and institutional reorganization or role change to enable consistent delivery of SST in the community; education and training of midwives, and of malaria coordinators on SST data reporting; inclusion of SST indicators into health facility registers and reports, and in the national HMIS; sourcing of more sensitive RDTS and improved quantification and distribution; and sustainability of funding.

There were several study limitations. The health facilities selected to participate in the study using purposive selection may not have been representative of all health facilities in the two study districts, or in the rest of malaria endemic Indonesia. The results from this study have limited generalizability beyond the population sampled but efforts were made to explore a wide variety of attitudes and opinions by interviewing participants across different health provider cadres and across all levels of the health system. Within the study participants, the major themes were relatively consistent suggesting that the findings reported here represent the extent of, and major obstacles to, implementation of the SST strategy.

## Conclusion

This study highlights areas for potential improvement in the implementation of SST in West Sumba and Mimika districts in Indonesia, particularly with respect to SST provision at village level, to reduce the number of missed opportunities. Specific areas needed to expand the delivery of SST to village health posts, where most pregnant women receive antenatal care, will require the reorientation and training of health providers, ideally with the provision of more sensitive RDTs that can detect all species, stable storage and supply chains for use in remote areas, improved data capture and reporting, and specific district budget allocation for malaria in pregnancy alongside more advocacy at district, provincial and national levels.

## Additional file

**Additional file 1.** Quotes from in-depth interviews with health providers on the feasibility of SST by theme and sub-themes. Quotes from in-depth interviews with health providers on the feasibility of SST programme in Eastern Indonesia.

## Abbreviations

ANC: antenatal care
DHO: District Health Office
DP: dihydroartemisinin–piperaquine
IDI: in-depth interview
RDT: rapid diagnostic test
SST: single screening and treatment in pregnancy
HMIS: health management information systems
