# Supplementary material for: Evaluation of the national policy of single screening and treatment for the prevention of malaria in pregnancy in two districts in Eastern Indonesia: health provider perceptions
Source: Malar J. 2018 Aug 24;17:309. doi: 10.1186/s12936-018-2426-y (PMC6108151; doi:10.1186/s12936-018-2426-y)
Supplement: Supplementary file 1 — Additional file 1. Quotes from in-depth interviews with health providers on the feasibility of SST by theme and sub-themes. Quotes from in-depth interviews with health providers on the feasibility of SST programme in Eastern Indonesia. [file 12936_2018_2426_MOESM1_ESM.doc]

Additional File 1: Quotes from in-depth interviews with health providers on the feasibility of SST by theme and sub-themes

| **Area** | **Themes/Sub themes** | **Quotes** |
| --- | --- | --- |
| **Acceptability** |  | **To what extent do health providers accept SST?** |
|  | **Of SST control of malaria in pregnancy** |  |
|  | 1) SST is good because it is important to detect malaria early in pregnancy, pregnancy is risky time | “I: In your opinion, is screening as the best way for handling malaria in pregnancy? R: In my opinion, screening is the best method. Every pregnant women should be screened.” Malaria coordinator, Sumba  “I: Do you think that the malaria screening program is the best method for malaria prevention in pregnancy? R.2: Yes, I think Malaria screening is the best method for malaria prevention in pregnancy. I: Why? R.2: It is because in Timika people already have immunity for malaria so although the patient has no symptom but actually the patient has parasite inside her/his body. It’s quite different with the outsider. We have found some cases like that. For example, the patient has no symptom but the screening result is malaria mix. So I think screening is very important.” Village midwife, Mimika  “I: Right. Do you think that this single screening and treatment is the best approach? R: Yes. I: Can you tell me more about it. R: It’s a – I mean, to perform a screening test to a pregnant women is a good thing because we are able to detect earlier. Besides, because this is a pregnant woman that is infected with malaria, definitely would put her pregnancy at risk, because this is malaria. “ Midwife coordinator, Mimika |
|  | 2) SST should be continued | “I: Do you think this screening is good enough to be done? R: I think this is good, it is important to be done, because the malaria cases are so high in this place. And it is also important for the babies’ health. I: Should it be changed? R: No, it shouldn’t. It is better to be continued.” Malaria coordinator, Sumba |
|  | 3) SST is a good policy but only if it is in fact being carried out and the quality is improved | “I: Is the malaria screening test on pregnant women without symptoms has been the best choice according to your opinion? R: If we want to call it best I don’t think it’s appropriate in this case, since this is one of the instrument or a way to detect malaria faster in pregnant women. The motto is the faster the better (early diagnosis and prompt treatment). It’s good when you actually do it, but whether it’s really being done is unknown. I think this is a good policy, but the quality needs to be increased and also the power behind it. The workers also need to be trained.” DHO, Sumba |
|  | **Of RDTs vs microscopy** |  |
|  | 1) RDTs are not always accurate | “I: In your opinion, to diagnose it, one should use RDT or microscopy? R: In my opinion, one should use the microscopy, since with microscopy, we can see and differ the parasites directly.” Lab technician, Sumba  “I: You said that you did first malaria screening at Puskesmas and follow up Malaria screening to pregnant woman at home, right? What method did you use to do that screening? RDT or slide? R: So far we never get accurate result from RDT. Maybe it was because the technique of blood sampling so for 1 patient we could get 2 results, negative result with RDT but positive result with slide. That’s why I prefer to use slide.” Malaria coordinator, Sumba  “I: After they done, do you think RDT is still feasible to use? R: Uhh, you mean Screening test by using RDT kit… if there is no other appropriate tools to perform the screening test then using RDT kit is our last option to take. From the effectiveness and specification, RDT is not as good as DDR. DDR has become our gold standard.” Doctor, Mimika |
|  | 2) RDTs are easy to use and a good alternative if there are no lab services or for use in the field | “I: Do you think that screening – using RDT method – is the best approach? R: I don’t think so. I hope there could be an RDT kit procurement so we can perform a test at the Posyandu. Sometimes we had a pregnant woman with malaria symptoms that we had to refer her to Puskesmas. It is fine if it is not that far from the house, but if it is far from the house then there could be a transportation issue. I hope Eijkman Institute can procure RDT so we can use it at the Posyandu.” Village midwife, Sumba  “I: in your opinion, do you think RDT is a proper tool to diagnose, to strengthen the diagnosis of malaria as an alternative to microscopic? R: I think it’s... it is proper, proper enough to use. First, it becomes handy during power down, as an emergency during power down. It’s pity to tell the sick patient to go home just because the electricity is down or that we don’t have slides, so that tools is really helpful.” Lab technician, Mimika  “I: So you mean RDT is only complement for Microscope? R: Hm..um (Yes). I hope it will be possible in the future. But now we have limited laboratory analyst at Puskesmas and the microscope method takes much time. In the other hand RDT is quite simple to be done and almost health worker or people can be trained to use it. But the problem is the RDT result is not as accurate as microscope. That’s why I said that RDT is only for alternative tool to confirm diagnosis of Malaria. We need accurate result before giving drug to patient. In my opinion microscope is better than RDT.” Midwife coordinator, Mimika |
|  | 3) RDTs are useful but we may still need to confirm result with microscope | “I: What do you think about the use of RDT to diagnose the patient? If there is a symptom RDT is used, and if there is not, then screening. To replace slide with RDT, do you think that is a good idea? R: According to me, we use RDT, but if the fever is serious then we should confirm into the lab. That’s what I think. I have once had a discussion in a meeting in Jayapura. We were given the explanation of doing so. We need to see the level of the sickness. If it is serious then we need to have the lab examination as well but we always use RDT at the beginning. That is like the temporary diagnose.” Midwife coordinator, Mimika  “I: If the pregnant woman come to Posyandu, what do you use to check her malaria? R: At ee… Posyandu we use RDT because what is?... but the pregnant woman could be asked to go to Puskesmas for DDR examination or maybe eee… It’s because sometimes we doubt the RDT result” Doctor, Mimika  “I: How if you find the pregnant woman with malaria symptom at village but the RDT result is negative? Would you ask her to come to Puskesmas on the following day? R: Yes, I would. The patient is suggested to come to Puskesmas.” Midwife coordinator, Sumba |
|  | **Of DP** |  |
|  | 1) Most participants reported no challenges with DP use | “I: About darplex, do you have any problem in using darplex? R: So far, nothing. I: No complaint from pregnant women? R: Nothing.” Malaria coordinator, Sumba  “I: Any problem in DHP prescription? R: So far, there is none, DHP is tolerated by most people and has minimum side effect. So far, it is seldom to have nausea complaints from the patients. Patients feel more comfortable and quicker to recover after taking DHP.” Doctor, Mimika  “I: How about Darplex? Any complain? R: I think there is no complain about Darplex. Ee… For Darplex, we ask the patient to take it for 3 days only. Darplex is a new drug for Malaria. It is not like Kina or Chloroquine that should be taken longer than Darplex. Mostly, after taking Darplex for 3 days, the patient looks well and fresh. That’s why they like Darplex. Actually, Darplex also has side effect but because the patient has to take it for 3 days, the side effect becomes less. The dose of Darplex is depending on the body weight. The patient could take 3 tablets at once per day but so far I seldom hear the patient complain about this OAM.” Midwife coordinator, Mimika |
|  | 2) DP was not always immediately effective | “I: What about the drugs used by health officers? Is there any problem about DHP or Darplex? R: For Darplex, in recent few days, there have been reports, several times that it is failed. The patients come to check up on them and they are given the drugs and they still come in the fourth days and they are still positively infected, but the rate was less positive for microscope. But we usually ask them to make the letter checks. And then they can come to check up for the second time and I am grateful that they are no longer infected in the second check up.” Malaria coordinator, Mimika |
|  | 3) DP can produce mild side effects in some women (nausea, vomiting, dizziness) | “I: do you have any obstacles in using darplex as a medication? R: The obstacle is stomach disorder sometimes occur in pregnant women. I: The side effect? R: The side effect of the drug may cause a nausea, because it is a large amount of drugs that has to be taken, it is then becoming an obstacle.” Doctor, Sumba  “I: Now we are talking about medication. Are there any obstacles for the use of darplex/piperaquine in this puskesmas? R: There is no obstacle so far. However, sometimes pregnant women feel faint, dizzy, and nauseous. So, we advise them to eat a lot, drink sugar water a lot, then take rests a lot. That’s it!” Midwife coordinator, Mimika  “I: How is the use of DHP/darplex? R: There is no problem! However, the pregnant women get nausea when consume, so it’s sometimes not finished. We give comprehension to finish the drugs. Health workers motivate and follow up as well.” Malaria coordinator, Sumba |
| **Demand** |  | **To what extent is SST perceived to have positive effects, to be used, and demanded by PW?** |
|  | **For SST by pregnant women (perceived by health providers)** |  |
|  | 1) Awareness about screening is low and some pregnant women don’t go for screening despite being advised e.g. transportation costs | “I: So if there is pregnant woman who comes for the first visit, how will you know whether she has Malaria or not? R: We ask her to have malaria examination at another health facility but sometime the pregnant woman doesn’t want to go. Now, we can do Hb examination for pregnant woman because we have bought the tools by the JKN funding. But for malaria examination, we ask the pregnant woman to go to another health facility but sometime she refuses it.” Village midwife, Mimika  “I: In your opinion, if the pregnant women are advised to visit puskesmas or hospital, could it be done? R: I think there is no an obstacle, because all services are free. They probably need to pay the transportation only. I: If the pregnant women come to posyandu, and there is tool for blood testing, so they would be referred to puskesmas, who would pay the transportation? R: There is no funding for it. The pay it themselves. BOK budgeting is for the transportation of health workers only. From the last 4 years, every pregnant women have been giving IDR. 250,000,-, and it’s included transportation to puskesmas.” Midwife coordinator, Sumba |
|  | 2) Lack of motivation to attend health facilities | “I: What kind of drugs you usually prepared? Are Darplex and quinine available? R: No. It should be by screening, we cannot ensure it immediately. I: How do you predict or measure the amount of drugs needed for prevention and treatment for malaria? R: There are only a few pregnant women. For it is a rare case.” Midwife coordinator, Sumba  “I: Is there any other activity, besides screening, to prevent malaria on pregnant women? R: Besides screening, we also have communication and counselling with the pregnant women, because they are unwilling to come to Puskesmas, unless when they are sick. So, they are recommended to come here so that they understand about the danger of malaria, even it could cause miscarriage. That’s all we do, because we cannot changed their lifestyle in an instant.” Malaria coordinator, Sumba |
|  | 3) Women question why they are being tested when they have no complaints | “I: What do you think if for every visit of a pregnant woman, she would receive a screening test? R: To perform a screening test – it could be done, but many women are not comfortable being punctured all the time. Sometimes a recipient refused to be punctured because there are no symptoms present, even for the first visit. So it’s more about recipient’s comfort, if she’s willing, but if she refuse to then there’s no way we would force her. Although there are explanation prior to the test, but most of the recipients are like ‘I don’t want to, I’m not feeling any fever’”. Midwife coordinator, Mimika  “I: There might be some alternatives to do screening test on the patients. Like, only in the first visit, in every visit or what? R: Perhaps it is quite hard to do screening in every visit if there is no illness complaint. The patients are usually reluctant to be examined over and over again because they are often scare if there is something wrong with them. So, the willingness to check up on themselves is decreasing over time. But now, I think it is going better.” Midwife coordinator, Mimika |
| **Implementation** |  | **To what extent is SST being carried out in health facilities?** |
|  | **SST** |  |
|  | 1) PW are being screened for malaria on their 1^st^ ANC visit (regardless of symptoms) | “I: How is the implementation of the screening test here Doc? R: Screening test for a pregnant women is running here. So for every pregnant women that get in touch with us either in Puskesmas or general clinic or KIA we tested for malaria. Even if they don’t have any symptoms but we still perform the screening test on them.” Doctor, Sumba  “I: Is there a new policy for malaria in pregnancy control? R: New policy? I: Or a new program from DHO or puskesmas? R: We emphasize the pregnant women who visit ANC at first to be screened. That policy is from DHO. I meant every pregnant woman have to be screened. That’s it!” Midwife coordinator, Sumba  “I: So that means... for every first visit, the patients will be tested, screening test? R.2 : Yes.. if... I: is it the first contact with the health workers? R.2: Ee…..in Posyandu it’s.... I: Then if a pregnant woman come with symptoms, she’ll be examined again? R.2 : Yes.. if.. if she is here.. she is here for treatment in... Pustu and in Posyandu, for every first visit of pregnant woman must be tested for malaria.” Village midwife, Mimika  “I: Okay. Ee… as a midwife who works here, do you usually do malaria screening for pregnant woman in the first ANC visit at Puskesmas? R: Yes, I do. All pregnant women have been screened for malaria in the first visit of ANC. Beside that the pregnant woman gets bed net in the first visit of ANC. I: in the first visit....? R: Right. In the first visit at Puskesmas, we always do malaria screening for pregnant woman.” Midwife coordinator, Mimika |
|  | 2) After the first visit, they will only be screened for malaria if they present with symptoms | “I: Do you usually do the screening test on all the pregnant women on their first visit? R: Yes, on their first visit. After that, if only they have the malaria symptoms then they will be screened again.” Midwife coordinator, Sumba  “I: So if the pregnant women came by without any symptom, you would not do any examination. R: Yes, if she was not K1, but she is K1 if she had symptom or not they would still get examined but if they were K2,K3,K4 we’ll examine if they have symptom like shivering or fever. I: so if a pregnant woman came without any symptom you will not examine her? R: if they have symptom then we’ll do the examination but if the K1 were standard procedure even if she has no symptom there will be a screening examination as usual.” Village midwife, Mimika  “I: For the second contact with pregnant women, does she need to be screened by AMC or not? R: Is she? I: if she come again for controlling. R: We check again… Oh no! We will only check if there is symptoms. If she already had malaria, and come for malaria control then we will check again. If she doesn’t come for malaria control, we only do screening if she has symptoms. I: Oh, based on symptoms? R: Yes.” Head of puskesmas, Mimika  “I: I noticed that at the next visit, it would be done only if there are symptoms occurred? R: yes, the first time is a must. And then after she receive ANC and she has medical complaints, even though at the first visit she has been examined, she has to be re-examine if she has any signs or symptoms and of the result turn out to be positive then would be proceeded to therapy, we have the standards.” Midwife coordinator, Mimika |
|  | 3) SST is reportedly not being implemented at all or not consistently | “I: So basically this hospital, doesn’t do malaria screening test and only the one with symptoms will be tested with malaria screening test. R: Yes. So if a pregnant woman came with a symptom of fever, anaemia, and especially if this woman is from Lamboya or another endemic area, we always test her for malaria every 6 hours. As well as if the pregnancy is going for well over 9 months and the child is born, we shall also take the child’s blood sample to be tested. Just in case the baby is infected by the mother.” Lab tech, Sumba (RSUD)  “I: So people who develop symptoms are the only people who are being examined and being given malaria screening test? What about the ones with no symptoms? R: Yes only the ones with symptoms, the ones with no symptoms are not recommended.” Midwife coordinator, Sumba (RSUD)  “I: the malaria screening test, malaria in pregnancy in particular, is it perform here in Posyandu and Pustu? R: The what? I: Malaria screening test in... R: beside the... microscopic? I: yes, especially for pregnant women in their ANC visits. R: It’s very rare in here unless the patient is already sick or having a symptom then we’ll examine her. I: And so does pregnant woman? R: yes all type of patient I: There is no screening test either in pustu and posyandu then? R: No there is not….I: for example, there is a visited pregnant woman for the first time in puskesmas. Whether she has the symptom or not, would she get a malaria in pregnancy screening test using the RDT? R: No, only if the patient has the symptom.” Pharmacist, Sumba  “I: Relating to the malaria prevention program how if the pregnant woman gets malaria screening in the first visit of ANC? R: E’e..(Yes). I: The first time… R: E’e..(Yes) I: She comes here for the first time and gets malaria screening to see… R: If there is no clinical sign of Malaria, the pregnant woman will not be screened. But if she doesn’t have bed net she will be given bed net for malaria prevention…..I: I see. Ee… There is national program that said all pregnant women who come to health facility for the first ANC visit has to be screened for malaria. R: Oh… If that so I think that program has been implemented now but as I know previously there was no program like that. However if there is instruction like that I’m sure that the midwife follows the instruction. I: It’s because you have more concern to store room thing or procurement, right? R: I have been working since 2005 and as I know previously they didn’t do screening if the pregnant woman didn’t have symptom. But as long as there is instruction to screen all pregnant women in the first visit even though they don’t have symptom, I think that instruction has been applied now.” Head of drugstore, Mimika |
|  | **SST at health posts/village level (posyandu, pustu)** |  |
|  | 1) Pregnant women are not being screened at health posts | “I: Why don’t you screen the pregnant women (K1 murni)? Do you have any reasons for it? Are there any instructions or programs from puskesmas? R: Since there is no in posyandu. That’s it! I: No, I meant from the program, isn’t done in posyandu? R: Seems nothing. So, we just immunize them in posyandu. Immunization, side dish supply, and ANC. I: So, there’s nothing for malaria screening test? R: Nothing!” Village midwife, Sumba  “I: When you run out of RDT at Puskesmas, what did the midwife use to do malaria screening to pregnant woman at Posyandu? R: As I said before, we never do malaria screening at Posyandu. I: Never? R: Never. I: Until now? R: Yes. So please don’t ask about that many times because we never do that at Posyandu.” Malaria coordinator, Sumba  “I: Right. Do you think where should screening test be performed? . R: E..ee….. I: Is it in Posyandu…or in Poskesdes or in Polindes or should it be in Puskesmas only? R: to perform at all of those would be good……. I: all of them..? R: in Puskesmas, the screening test has been covered by the Puskesmas, I mean without Stopmip, Puskesmas already performed it, while posyandus and Pustu, it’s not available there.” Midwife coordinator, Mimika |
|  | 2) Only carried out at health posts if RDTs are available | “I: So the screening and the treatment are implemented only in Puskesmas, not in Posyandu? R: There is also SF tablet distribution, such as vitamin in Posyandu. I: But there is no screening or blood sampling? R: Malaria? No. Unless if the RDT is available. Now the RDT is no more available. I: So previously there was screening activity in Posyandu? Using RDT? R: Yes, it was. I: Now it is not available? R: No, it isn’t.” Midwife coordinator, Sumba  “I: You mean the pregnant woman? R: Yes. The pregnant woman should be screened for Malaria even though she has no complaint. We have done this program from the beginning in 2014. Malaria examination and HIV examination is a must for pregnant woman unless at the place where we can’t do that examination like Posyandu. At Posyandu we must do malaria examination with RDT to pregnant woman who has complaint. But if there is no RDT at Posyandu, we will ask the pregnant woman to go to Puskesmas.” Midwife coordinator, Mimika |
|  | 3) Pregnant women are told to go to health centres for screening | “I: Have you ever had experience to do malaria screening at Posyandu? R: Screening at Posyandu? I: Yes. For pregnant woman at Posyandu. R: I never do that. I: Never? R: Never because we don’t have any equipment to do that. I: So you always ask the pregnant woman to…? R: I ask them to go to Puskesmas.” Village midwife, Sumba  “I: Ee… which units of Puskesmas do you applied single screening and treatment program? Do you apply it at Posyandu or? R: we haven’t applied that program at Posyandu yet. I: Why? R: It is because if we don’t have RDT so there will be no reason to bring anti malaria drug at Posyandu. That’s why we ask the pregnant woman to go to puskesmas for malaria screening. I: So every pregnant woman who comes with symptom at Posyandu will be asked to go to Puskesmas, right? R: Right.” Midwife coordinator, Mimika |
|  | 4) Screening is being done at health posts using RDTs | “I: Does the midwife screen the pregnant woman who comes to Posyandu for the first visit? R.2: Yes, she does. I: At posyandu? R.2: Yes.” Village midwife, Mimika |
|  | 5) In some village settings, only symptomatic women are being screened | “I: Is screening or blood taken done in posyandu? R: Not every month. It’s according to the program. I: What do you mean? What program? R: We decide 20 pregnant women as samples. I: Oh, from malaria program coordinator? R: Yes. I: It’s decided how many pregnant women for screening? R: Yes. I: For others? R: For others who have symptoms, we refer them to puskesmas for blood taken. That’s it. I: So, for pregnant women with clinical symptoms only? R: (nod).” Village midwife, Sumba  “I: do you have experience or in your observation when you were in PHC, every pregnant women who first come and get the malaria screening immediately or how? R: I do not really know in PHC. I: how about here in Pustu? R: as long as I am here, we never did it without complaints. I: So symptoms. R: yes. I: And then get the examination? I: Have you ever get the information about pregnant women who come for the first time need screening…with or without symptoms? R: I have heard about it, but in Timika PHC, in treatment room in delivery room, we do not do the screening, that the job of ANC. Here, we do not have many pregnant women, we can count their number.” Village midwife, Mimika |
|  | 6) Challenges with implementation at village level:  a) Limited RDT stocks or complete stock outs  b) Lack of staff (trained staff, lab technicians) | “I: I see, so the screening is done on every patient only in their first visit, so, is it done in Posyandu or Puskesmas? R: Actually, the screening test is done in Posyandu, but the availability of RDT kit is still our main problem. The RDT kit logistic is limited or simply not provided, so the patients need to wait till they have a chance to do that in Puskesmas. Or, if the RDT kit is available, then the patients can be screened in their own home. We give them the service not only in Puskesmas but whenever we have a chance to pay a visit on the field with the checking equipment. But for those who are not serviced in their own home, will be serviced in Puskesmas.” Midwife coordinator, Mimika  “I: So you never done screening to pregnant woman at Pustu? R: I think so. I: That is because the tool is not available here? R: Our problem is the tool is not available but when we have the tool, no pregnant woman came here. One day I brought Doppler, Blood pressure machine and measuring tape for LILA (Upper arm circumference) but no pregnant woman came on that day. I: Is RDT available at Pustu right now? R: No, it isn’t.” Village midwife, Mimika  “I: From 10T that have to be done in ANC, then added malaria testing, what do you think? R: The obstacles are about time and logistic. It’s probably done in puskesmas, and should be used RDT in posyandu, and sometimes not available.” Midwife coordinator, Sumba  “I: Now there is more... How far do you think you can run this program? R: For the screening, I am pretty sure that the workers there are skilful enough… however, the availability of the logistic is still the current problem we face. They will do the screening if the logistic is available. But, like I told you before, the workers in suburb are not always available. Moreover in the hinterland, they are usually in the village for 3 months and will be in town for a month.” Malaria coordinator, Mimika  “I: Overall, what is it needs to be fixed for the better future? R: Human resources need to be highly noted. The stuff needed for the programs, such as the logistics availability, medicines, and other stuff require good planning. Other thing that is important will be the welfare of the workers.” DHO, Sumba |
|  | **RDT availability** |  |
|  | 1) RDTs have never been used; are not available | “I: Have you ever used RDT? R: The whole time I am assigned here, I have never done a test using RDT. I: Have you seen RDT before? R: I did a test using RDT during my training in Sumba Foundation. But when I came back to work, we have never done a test using RDT.” Lab tech, Sumba (RSUD)  “I: Is RDT available here in KIA? R: No. I: No? R: RDT is not available because we have laboratories available from morning until evening.” Midwife coordinator, Mimika  “I: Is the positive result come from RDT or Microscope examination? R: in this book? I: Yes. R: Microscope only. I: Oh…microscope only? R: For RDT eee... We are not used to use RDT, very rare.” Midwife coordinator, Mimika |
|  | 2) Current stock outs for RDTs and previous stock outs across facilities/areas | “I: What’s the tool for screening? R: The test is in the laboratory with microscope. I: Oh microscope? R: There is no RDT here so far. I: Oh… how long it hasn’t been here? R: It’s already been 4 or 5 months. During at the end of last year and early this year until now, it’s not been here.” Midwife coordinator, Sumba  “I: Do you have the stock of RDT now? R: No.” Village midwife, Sumba  “I: Have this Puskesmas an RDT? R: RDT is on shortage now. I: On shortage. For how long has it been? R: E…probably just for a month. I: What caused it to be on shortage? R: The warehouse staff haven’t made the request order yet, because it is done every three months. It’s just not requested yet.” Doctor, Mimka |
|  | **Antimalarial prescription at different facility levels** |  |
|  | 1) Antimalarials not available at health posts; pregnant women must go to health centres to receive treatment | “I: If you found the pregnant woman with Malaria infection at Posyandu… R: Yes. I: Who would give the treatment to the patient? R: If we found the pregnant women with malaria infection at Posyandu, we would refer them to Puskesmas so the Doctor could give them treatment.” Midwife coordinator, Sumba  “I: So, if the pregnant women who come to posyandu and complain about the symptoms of malaria, they would then be referred to puskesmas or? R: Yes, we refer them to puskesmas. I: Aren’t there any drugs in posyandu? R: Nothing! We just bring paracetamol, anaemia tablet, that’s it. As I know, other friends do it as well.” Village midwife, Sumba  “I: How about the malaria treatment at Pustu if there is patient has Malaria? R: The patient will be referred to Puskesmas because Pustu is not far from here. I: Oh Pustu is near here so the drug will be given at Puskesmas. R: Yes.” Midwife coordinator, Mimika |
|  | 2) Treatment prescribed by a doctor | “I: what happened if a screening test performed in Posyandu acquire a positive results, what would be the therapy, by whom? R: At certain posyandsu, doctor participates, and when we participate, patients would be directed to us, and if we don’t participate, patients would be directed to Puskesmas.” Doctor, Mimika |
|  | 3) Doctors and/or midwives provide pregnant women with prescription (at ANC) for treatment of MiP; women collect antimalarials from pharmacy | “I: who usually prescribe the medicine? R: well, all of us… prescribe. The medical prescription that we give is based on the standard procedure, but it’s the pharmacy that issue drugs. So we prescribes, patient can take the drugs at the pharmacy. I: So who prescribes? R: the KIA? I: The midwives? R: Yes. I: And then take it to… R: Take it at the pharmacy. I: Take it at the pharmacy… R: yes.” Midwife coordinator, Mimika  “I: And for malaria in pregnancy? Is there any specific program? I mean, is there any separation for the malaria in pregnancy? R: Aa.. it’s on the reporting. All drugs are stored in pharmacy. So the midwife would prescribe and patients may take their medicine at the pharmacy. Midwife would not give the medicine; i would not give the midwife the drug stocks. Never and never will.” Malaria coordinator, Mimika  “I: It is based on the prescription from the doctor. R: prescription from the doctor? I: yes. R: then proceed to the pharmacy. R: Proceed to the pharmacy. I: the pregnant women come to the pharmacy by herself? R: Yes. Iya, pregnant woman come to the pharmacy by herself taking the prescription with her, we prepare the medication then give it to her.” Pharmacist, Mimika |
|  | 4) Antimalarials cannot be prescribed without confirmation from a diagnostic test for malaria | “I: You don’t give anti malaria drug? R: If the pregnant woman has malaria positive we will give her anti malaria drug. If she doesn’t have it we afraid to give that drug. I: How if the pregnant woman comes with symptom? R: If she comes with symptom but she doesn’t bring the examination result that shows malaria positive, we will not give the drug because the point is the drug will be given if the examination result is positive.” Village midwife, Mimika  “I: So when you did home visit, what kind of drug did you bring? R: Iron tablet, Calcium, Vitamin B complex, Vitamin C. I: How about anti malaria drug? R: Oh I don’t bring that because we are not allowed to give that drug randomly. The patient should get blood examination completely. Although we have the drug but we are not allowed to give it randomly.” Village midwife, Mimika |
| **Practicality** |  | **To what extent is SST being carried out using existing resources** |
|  | **RDT vs microscopy** |  |
|  | 1) Microscopy is main method of screening at health centres | “I: Single screening and treatment for pregnancy has to use RDT, how is it implemented in this puskesmas? R: We use both RDT and slide. I: Why? R: Since we have lab technician with good qualification, so we use slide. RDT stock is limited as well, so it would be distributed to posyandu only. If the midwives are not confident with the result of RDT in posyandu, they would refer to here. I: Have you experienced the pregnant women who are screened by using RDT here? R: No. We use slide. I: Blood slide. So, you have never used RDT here? R: Never. We use microscope.” Doctor, Sumba  “I: Where do malaria examination occur? R: at the laboratory. I: For pregnancy? R: Still at the laboratory. I: All are performed at the lab, none at the KIA? R: No, the lab. I: What tools do they usually use? R: They use microscope. I: microscope, never did they use RDT? R: No RDT is meant for Pustu. Even under certain circumstances, they would not use it.” Malaria coordinator, Mimika  “I: is it done in this Puskesmas? R: Yes, it is. I: What they use for screening in this puskesmas? R: They use microscope for screening. I: Using microscope? R: Right. Previously they used RDT even though they have limited stock of RDT but after they got microscope they use it.” Midwife coordinator, Mimika |
|  | 2) Microscopy is used primarily but sometimes RDTs are used when the electricity is out or lab services are not available | “I: What method do you use for a screening test? RDT or microscopic? R: I used both. If the power shut down, I use RDT first but I still use microscopic examination later. Because I’m not easily satisfied by the result of RDT. I have to ensure it with microscopic examination. I: I mean do you use both? R: Yes for malaria in pregnancy screening test. I: What is the method of screening test you use to test the pregnant women in their first contact with the health care provider? R: Micros… I mean slide examination. I only use RDT when the power shuts down, but I rarely use RDT.” Lab tech, Sumba  “I: When the pregnant woman comes here, what tool do you use to do the examination? R: What examination? I: Blood examination for malaria? R: Microscope. I: How about RDT? R: As long as the electricity is available we use microscope but if the electricity is gone or the analyst doesn’t come, we will use RDT. I: RDT? R: Yes. But as long as the analyst and the electricity are available, we use microscope.” Pharmacist, Mimika |
|  | 3) When available RDTs are used for screening at health posts/sub health centres | “I: Do you only use slide method for malaria examination at puskesmas? R: Yes, I do. But we used RDT at posyandu.” Malaria coordinator, Sumba  “I: Do they use RDT for malaria screening? R: Yes, they use RDT. However, for pregnant women who come to puskesmas could be tested by using either RDT or slide. I: Based on the guideline, how? R: For screening in pustu and polindes, they use RDT. However, in puskesmas they use slide.” Malaria coordinator, Sumba  “I: So what equipment do you use for screening? R: In this main puskesmas, we have microscope so we prioritize using it, but when we are at the field for Posyandu we use RDT, but we still take the blood samples.” Doctor, Mimika |
|  | 4) RDTs are often administered by midwives, or lab technician (reported at one location) | “I: What will be the challenge from Pustu? R: Instruments. I: What instruments? I: Do you mean for malaria examination? R: We usually use this, stick (while showing RDT on her desk) I: RDT? R: E.. yes. I: Do you have microscope here? R: No, we do not have analysts. I: So do you use RDT? R: Yes. I: Have you ever get workshop to use RDT? R: Not for me, but my other friends, yes. I learned by myself.” Village midwife, Mimika  “I: But if the equipment is available? R: We would give the healthcare. I: Using Rapid test? R: Rapid test. I: Who perform the examination? Are you? R: Yes, midwives.” Village midwife, Mimika  “R: In posyandu we have this established procedures, also known as protap. We don’t take drugs to Posyandu with us. Just in case a diagnose shows that there is a patient infected with malaria we would suggest her to go to Puskesmas. I: What tools do you use to diagnose. R: We use RDT to diagnose in Posyandu, but we are a team in Posyandu, so we are not equipped with RDT, but the laboratorians are. So the laboratorians will perform the examination, while we focus on the pregnancy. We check for her heart rates, gestational age then proceed to the lab section. I: But do you take the RDT stock with you to Posyandu? R: RDT -- Laboratians do, the midwives don’t. I: Everytime you go to Posyandu? R: Everytime we go down to Pusyandu, midwives never take RDT with them, it’s the laboratorian’s duty.” Midwife coordinator, Mimika |
|  | 5) RDTs are not being used at the health posts | “I: So, the midwives are not informed how to use RDT for malaria screening test for pregnant women? R: There is no RDT. I: So, based on your experience, have you done it? R: I’ve never done it since 2012. I’ve only done immunization. I: Those who are handled by you, have you ever done by using RDT? R: No!” Village midwife, Sumba  “I: Why did not you take RDT kits with you to Posyandu? R: that because -- I don’t know, we were always told that RDT stock is limited, and because malaria is diagnosed using microscope at the laboratory instead of using RDT. I mean RDT is less accurate, more microscopic examinations are using microscope. There’s no way we would take microscope to Pustu, to remote areas. Until this moment there are still no RDT available. Perhaps the same question should be asked to the midwife coordinator, perhaps she would inform that to the authorities, which perhaps would supply RDT. Because probably RDT’s stock are limited everywhere. I: So since you started working in this Puskesmas, you never brought RDT kit to Posyandu? R: Never.” Village midwife, Mimika |
|  | **SST at different levels of health facility** |  |
|  | 1) SST should be done at health posts as they are more accessible | “I: Do you think RDT should be done at the posyandu or should the pregnant woman referred to the Puskesmas? What do you think? R: RDT is meant to be carried around, to Posyandu. Puskesmas already has microscope. RDT made it possible to perform diagnose everywhere. So RDT is good for the remote posyandu, while puskesmas already has microscope.” Midwife coordinator, Mimika  “I: Do you think which one is better to perform a screening test at? Is it in Posyandu or in Puskesmas? R: I prefer in Posyandu, mbak, because it is more concentrated. The problem in Puskesmas for pregnant women is the distance between their residence and Puskesmas. Those who live far from Puskesmas are very seldom to come to Puskesmas, they go to Posyandu instead. I prefer in Posyandu, because we got to interact with people.” Village midwife, Mimika |
|  | 2) SST should be done at both health posts and health centres | “I: do you think which one is better to perform SST, at the Posyandu or at the Puskesmas? R: Because there are more pregnant women in Posyandu, it is better to perform at both places. Posyandu is only conducted for two to hours whereas Puskesmas is always open, so to perform at both will be better.” Midwife coordinator, Mimika  “I: Right. Do you think where should screening test be performed? R: E..ee….. I: Is it in Posyandu…or in Poskesdes or in Polindes or should it be in Puskesmas only? R: To perform at all of those would be good…” Midwife coordinator, Mimika |
|  | 3) At health centres as they have microscopes and staff | “I: Which one do you think is better, RDT at the Posyandu, or to refer pregnant women to the puskesmas? R: I prefer to refer pregnant women to the puskesmas. In posyandu we only take blood sample, without knowing the result so they still have to go to the puskesmas if the result is positive. I feel pity for them if they had to go all the way from a distance house to puskesmas. So when we examine her pregnancy, we also perform a screening test on her, and the treatment as well. I: So you think it is better if pregnant women go to Puskesmas? R: Yes, just refer them to Puskesmas.” Village midwife, Sumba  “I: So you think the RDT examination can be done at Posyandu… R: We asked all pregnant women to go to Puskesmas for the first visit of ANC. I: So you think RDT has to be provided at Posyandu, right? R: I don't think so. I: You don't. Why did you say that? R: I said that because the puskesmas, the hospital and the Pustu are not far from Posyandu. I: Oh you think it is because the distance is not far, right? R: E’ee..(Yes).” Midwife coordinator, Mimika |
|  | 4) SST at all facilities is good if you have the resources | “I: So, in your opinion, where should this single screening (SST) can be applied? In what level? Pustu, IHC, PHC, or everywhere? R: Maybe it will be good to apply in those three places, but in Pustu, we do not have the facilities like in PHC, IHC usually has team from PHC so the facilities are different. I: Don’t you have an analysist here? R: No. We don’t. I: Do you have RDT? R: Yes, we do have RDT. I: So, for who can use RDT? R: for general screening for malaria. I mean everybody can use RDT. I: Do you have special test for pregnant women? R: No. We do not have, this we get from……” Village midwife, Mimika |
|  | 6) SST at home would be the best option | “I: According to you, where is the best place to perform malaria screening test? R: umm, according to me, the screening test for pregnant women is better if we also do sweeping as well. It means we visit their houses. And for doing the screening test I think it is good, just be more, more hm, more evenly done, more extend to the others… not only for those who come, to be examined here is rare.” Village midwife, Mimka |
|  | **DP for treatment** |  |
|  | 1) DP is prescribed for treatment in 2^nd^ & 3^rd^ trimesters | “I: What about the darplex? In what trimester it will be given? R: In second trimester. I: To upper? R: Yes.” Midwife coordinator, Sumba  “I: So what drug do they usually prescribe? R: Usually, let’s just say, in first trimester we would give quinine, second semester and above we would give DHP. I: Is it the blue drug? R: Yes. DHP.” Malaria coordinator, Mimika  “I: If the result is positive, what kind of drug do you give usually? R: OAM [Obat Anti Malaria = Anti Malaria drug]. I: OAM? R: But for less than 12 weeks of pregnancy, we give Kina. I: Mm… less than 12 weeks of pregnancy? R: Right. We use Kina for the first trimester of pregnancy and use OAM for another trimester of pregnancy. It is mentioned at guideline. I: OAM? R: Hm..um (Yes), OAM is Darplex. I: The drug with blue colour? R: Yes. We give that drug according to the body weight.” Midwife coordinator, Mimika |
|  | 2) DP stocks were mostly stable, but a few participants reported occasional stock outs | “I: So, do you have problems which are related to the drugs stock out, especially for malaria drugs, such as darplex and quinine? R: There is no problem for quinine, but we find for darplex. In the past, there was no darplex in drug store, and the doctor should change it with quinine.” Midwife coordinator, Sumba  “I: Have you ever run out of stock of Darplex and Quinine at Puskesmas? R: Yes, I have. We run out of stock of Darplex starting from end of 2013 until early 2014. Fortunately, we still had quinine at that time so we used quinine for Malaria treatment.” Malaria coordinator, Sumba |
|  | 3) DP is well tolerated and effective | “I: About darplex, do you have any problem in using darplex? R: So far, nothing. I: No complaint from pregnant women? R: Nothing.” Malaria coordinator, Sumba  “I: Any problem in DHP prescription? R: So far, there is none, DHP is tolerated by most people and has minimum side effect. So far, it is seldom to have nausea complaints from the patients. Patients feel more comfortable and quicker to recover after taking DHP.” Doctor, Mimika  “I: Ooo… how about the DHP? R: DHP is better than Kina especially for the side effect. I: But it will be given not in the first trimester of pregnancy, right? R: Right. After first trimester of pregnancy.” Doctor, Mimika |
|  | 4) Health provider concerns about completing doses of antimalarials; DP has shorter dosing regimen than quinine, could be better for compliance | “I: Do you think that this SST is the best approach? Or do you think there are other methods better than SST? R: I think it is better than what we used to have. At that time quinine took 7 days which was quite a long time. I: Quite a long time? R: Yes, I think this is better in term of patient’s obedience in taking medicines as compared to 7 days period. Patients want a quick recovery but not willing to take the 7 days period of taking medicine so sometimes they skipped days.” Doctor, Mimika  “I: So, do you think single screening and treatment like what I said before for the first time come pregnant women must get malaria screening with or without symptoms. Do you think this is going to be the best approach to prevent malaria or do you have suggestion on how to prevent malaria from pregnant women? R: This method is good, my concern is only that do they eat the medicine or not? Nayaro has the highest malaria case.” Village midwife, Mimika  “I: E’e. About the complaint from your patient after you give the drug. Is there any complain? R: Oh... There is no problem with that. We only… ehem what is it?… When we give anti malaria drug to patients, we always remind them to follow the prescription. The point is the patients have to be convinced to finish the treatment. Usually patients stop taking the next dose because when they take the first dose, they feel better and decide to not take the next dose. That matter can increase the risk of drug resistant.” Doctor, Mimika |
| **Adaptation** |  | **To what extent have changes been made to existing systems to implement SST?** |
|  | Replacement of microscopes with RDTs | *See quotes on availability of RDTs* |
|  | Antimalarials given by midwives in villages | *See quotes on prescription of antimalarials at different facility levels* |
| **Integration** |  | **To what extent has SST been integrated into the existing health system?** |
|  | **SST at health centres (current strategy)** |  |
|  | 1) ANC and lab work together in screening pregnant women for malaria | “I: Back to the coordination, the screening is done by whom? Whether it’s done by the midwives or health workers of malaria? R: Coordination among lab technician, malaria program, and midwife. If the midwives who screen, the report would be given to malaria program coordinator of puskesmas, it would then be forwarded to the district level. So, there is coordination among lab technician, malaria program, and midwife.” Malaria coordinator, Sumba |
|  | 2) Midwives carry out screening and malaria coordinator is responsible for reporting | “I: Do you have special division to facilitate and responsible for the cooperation between KIA and Malaria organizers? R: The Malaria organizers. He/she takes the responsibility for the reports and other related things. I: So far, is your cooperation with the malaria organizers going well? R: It is going well. I: No significant obstacle? R: Because we, all workers in Puskesmas always have a mutual cooperation.” Midwife coordinator, Sumba  “I: Would you like to tell me about your work and responsibility? R: Sure. As a coordinator I have to take care of all of things that related to KIA program and KB (Family Planning) program. I: Okay. Do you know anything about a new strategy of malaria prevention in pregnancy? R: Actually… ee… I think there is training about that but based on my experience there are many methods to prevent malaria in pregnancy for example bed net distribution. I: How about the malaria screening for pregnant woman? Do you know it? R: Yes, I do. Usually the pregnant woman should be screened first and pregnant woman would get bed net no matter what the result is, positive or negative.” Midwife coordinator, Mimika |
|  | 3) Antimalarials being given by midwives | *See quotes in* ***implementation*** *– antimalarial prescription at different facility levels* |
|  | **SST at village sites** |  |
|  | 1) Midwives request drugs and RDTs from health centre pharmacy/drugstore to take to health post/sub health centres | “I: Relating to the logistic that you need for Malaria in pregnancy program, like RDT, slide and drugs, where do you get those things? How do you get those things for Puskesmas? R: I get those from Pharmacy storeroom at Puskesmas. We just wrote our request o the paper and then give that to Pharmacy staff. As I know, The District health office distributes those things to Puskesmas through Pharmacy at every puskesmas. After the doctor prescribed the drug, the patient could take that drug from Pharmacy at Puskesmas.” Village midwife, Sumba  “I: For items distributed to posyandu, such as RDT, who is in charge of this distribution? R: Responsible for the distribution, usually before we leave for posyandu, we’ll check for this box and if it is empty, we’ll report it to the staff in warehouse and ask for one box of RDT before we leave for posyandu. The warehouse distributes. I: The warehouse? R: Yes. I: Then what happen if it ran out? R: Report it back to the ware house. Yeah, if it ran out we’d ask for it again from the warehouse.” Malaria coordinator, Mimika |
|  | **Quantification of supplies** |  |
|  | 1) RDTs/ antimalarials are ordered based on the monthly consumption reports by ANC | “I: In relation with logistics availability, how many RDTs, how many medications, how many curtains, how is the procurement? How to count the logistics need? R: Our policy is that all being handled by the pharmacy. So when the logistics come, everything being handed to the pharmacy. The program management requesting the pharmacy to be forwarded to puskesmas or the target. But sometimes to speed up the process, the distribution, the noting and administration process that sometimes being missed by the management. I: Who counts the demands? R: The program management based on available data.” DHO, Sumba  “I: How do you predict the number of drug, bed net, RDT, etc that you will need in one month forward? R.1: We make prediction based on the previous month report for example there is such a number of patient in the previous month so we assume that we will need more than that a bit for this month. I: Oh, you ask more for this month? R.1: Yes. So we make a request based on how many RDT or drug we used last month but for nest request if it is possible we ask more than that.” Village midwife, Mimika |
|  | 2) Orders are placed by pharmacy to DHO quarterly | “I: You ordered drugs from the health department? R: Hm.. um.. (yes), at the pharmaceutical warehouse. I: Oo.. pharmaceutical warehouse in Puskesmas? R: in SP2. I: Oo..SP2 belongs to the health department? Ooo.. I see. Then… R: We order the drugs every three months. And the drugs order controlled by malaria program, so the drugs are in the storage. I: Not ordered by the pharmacy? R: No, there is a malaria program, the drugs are kept in the warehouse. I: warehouse here? R: In the pharmacy, yes.” Pharmacist, Mimika  “I: Well, I want to ask about the stock of RDT. Where did you get RDT that you usually use at laboratory? R: The Stock of RDT at laboratory is only for villages. We have to make procurement to District health to get RDT. Then the district health will send it to GFK (District Pharmacy storeroom) before that RDT will be send to our pharmacy storeroom at Puskesmas. I: Did you make procurement by your self? R: Yes, I did. When we had no RDT left at laboratory, the Pharmacy coordinator sent new stock of RDT to laboratory.” Lab technician, Sumba |
|  | 3) RDTs are used for all patients/not exclusively for pregnant women | “I: So, as you the 50th RDTs are not for pregnant women only, but for general patients also? R: It’s for general, including pregnant women who come with clinical symptoms. But most of them would be referred to puskesmas to be checked, and it would be good for recording and reporting. It’s more about optimum services as well.” Pharmacist, Sumba  “I: so how did you count the RDT for pregnancy at the Lab? R: I did not count, I requested it. I: for example? R: For example, I have to supply each pustu with 100 stick of RDT kit, of that 100 sticks pregnant patients are in it. I: 100 pregnant women to be examined? R: Not 100 pregnant women, but of that 100 sticks it could be for general patients, it could be for pregnant women. And if it’s on shortage we may request it. I: Oh. So it’s not particularly meant for the pregnant women R: No. I: Just in general? R: In general.” Malaria coordinator, Mimika |
|  | **SST indicators into HMIS** |  |
|  | **Indicators collected by ANC at health centres & health posts** |  |
|  | 1) How many PW attended facility & how many receive malaria screening | “I: You always make reports to the district, so what are the indicators that you report, especially for malaria in pregnancy? What are the important points that you provide on the report? R: The number of pregnant women who have been screened in a month, then from the screening result, how many pregnant women who have positive. We also report what drugs that we give to them. That’s it! I: Is it separated? I meant there is a certain column for the number of malaria screening test. R: Yes, there is a column for the number of pregnant women who have been tested. For instance, from 10 pregnant women, how many positive, how many negative.” Midwife coordinator, Sumba  “I: How many pregnant women have been registered in this Puskesmas in this month? R: E..18 (18 people) I: 18? R: 18 consists of 8 pregnant women who are registered in K4 and 10 pregnant women who are registered in K1 kontak and K1 murni. I: Then ee… Have you screen all pregnant women that were registered in the first visit? R: Yes, I have. I: Where were they screened? R: At Puskesmas.” Midwife coordinator, Mimika |
|  | 2) Number of ANC visit when malaria screening was done is recorded | “I: do you separate the data of screening test at the first contact and at the next visit? R: Yes. I: At the report? R: Yes we have it written in LB3 book, this many of K1 visit, and then the next visit, it is separated.” Midwife coordinator, Mimika  “I: Relating to the number of visit of pregnant woman, do you classify that into 1st antenatal visit and subsequent antenatal visit? R: Previously… I: Do you classify that Malaria screening based on the number of antenatal visit? R: Yes, I do. We calculate the number of pregnant woman who got malaria examination at antenatal visit. For example there are 20 pregnant women at 1 posyandu, we have to classify into two groups, the examination with RDT and with slide. I: Do you mention about the number of visit? R: What visit? I: I mean 1st antenatal visit or 2nd ante natal visit and so on… R: Yes, I write the number of antenatal visit as well. I: You put it in the report? R: Right. I write K1 (1st antenatal visit) or K4 (4th antenatal visit) or the following antenatal visit.” Midwife coordinator, Sumba |
|  | 3) SST is recorded in the ANC booklet and/or cohort book | “I: So how do you make a report? R.1: We make a report for all pregnant woman visits. I: All visits? Each visit? R.1 : Yes. So every time the pregnant woman comes here routinely starting from the first visit until delivery visit, we record the data in this book (book means cohort book). I: Hmm... This is for the first visit? R.1: Yes.” Village midwife, Mimika |
|  | 4) Diagnostic technique (RDT or microscope) is recorded at some facilities but not others | “I: About malaria in pregnancy screening test, is there any recording about microscope and RDT usage? R: There is.” Midwife coordinator, Mimika  “I: You said that Malaria examination is done with Microscope and RDT. How do you know the mother is screened with microscope or RDT? Do you write it on the report book or is there any report for that? R: We have report for that. There is one book for malaria report here, like this (the participant shows report book of Malaria that lies on the table) but only positive result will be recorded in this book. I: Only positive result? R: E’ee...(Yes). I: Is the positive result come from RDT or Microscope examination? R: In this book? I: Yes. R: Microscope only. I: Oh microscope only? R: For RDT eee... We are not used to use RDT, very rare.” Midwife coordinator, Mimika |
|  | **Indicators collected at village sites** |  |
|  | 1) How many pregnant women attended facility, received screening, positive/negative results | “I: What points you put in your report? R: I have to report anything about the examination that I have done at village. For example, the number of pregnant woman who gets screening, the number of positive result and the negative result, the number of pregnant woman who gets treatment and the number of pregnant woman who doesn’t get treatment. That’s all. I: Do you mention about the kind of the drug too? R: No, I don’t.” Village midwife, Sumba |
|  | **Indicators collected by lab** |  |
|  | 1) Lab does not specifically collect data on PW, it reports general malaria data | “I: Don’t you make report about malaria examination in pregnancy separately? R: The malaria coordinator made special report about the malaria examination in pregnancy. In here I just made report about laboratory examination in general. I: Oh only in general version. R: Right. The Malaria coordinator made the report in detail so we could know the number of pregnant woman with malaria infection complete with the detail of pregnant woman identity, the husband’ name, etc. The point is I wrote the number of pregnant women or adult patient who had been examined for Malaria on the registration book complete with the date. So Malaria coordinator could calculate the number of malaria examination that was done at Puskesmas, including at KIA (mother and child health) room or out patient clinic and could make a complete report based on that book as well.” Lab technician, Sumba  “I: or perhaps there is a pregnant woman. How is the report? Where do you report it to, the malaria responsible person or the KIA? R: We make our report. They make their report too. Then we have the person in charge of malaria here that will sum up all the reports. I: What I meant was, if there is a pregnant woman come for a diagnosis, will there be any report in particular? R: No, it’s in general.” Lab technician, Mimika |
|  | **Health facility reporting** |  |
|  | 1) ANC collects data on SST, malaria coordinator compiles the report but collates all malaria data; (no specific report that links SST with pregnant women or ANC visit) | “R: Actually as the person in charge of malaria, I don’t know much, I’m just making the report. That’s all I do. That’s why if anyone asked me about mosquito net, I wouldn’t know, about anti malarial drug, pregnant women, I have no idea. Pregnant women are totally midwives’ matter. . they must have had the record. I: So the report at the other day was a P2M report in general? R: in general. Whether it’s a pregnant or non pregnant patients, it’s all incorporated, inseparable. I: But, the report for the pregnant women was empty, wasn’t it? (I mean the report of person in charge of malaria, at the column of pregnant women, was left empty) R: Yes.” Malaria coordinator, Mimika  “I: On the reports, cannot be seen the pregnant women at 1st visit have been tested whether it’s based on the symptoms or not. R: Cannot be seen in this report. Just for pregnant women in general, cannot be seen the trimester. About malaria screening probably is in P2 malaria. I: Do midwives have the data for pregnant women at 1st visit? R: Yes, it’s in here. I: It has to be related between K1 and malaria testing, but there is no on this report. R: Cannot be seen. This is the main format, and not written about malaria testing. It’s probably in P2 malaria.” Midwife coordinator, Sumba |
| **Expansion** |  | **To what extent has or can SST be scaled up and expanded?** |
|  | **Regular RDT supplies** | *No quotes* |
|  | **Health provider roles** |  |
|  | 1) Malaria coordinator is primarily involved in reporting on malaria in general; often has another role such as lab tech or nurse; sometimes directly involved with SST program | “I: Relating to your responsibility as a Midwife coordinator, have you ever coordinated with Malaria Coordinator to handle malaria examination for pregnant woman at antenatal visit? R: Sure. I have to coordinate with Malaria coordinator if I find the case. I usually refer the pregnant woman to malaria coordinator, who also works as a Laboratory technician at Puskesmas, to get malaria screening. Laboratory technician tells me the result and then I also coordinate with the Doctor to give treatment to patient. I: How was your collaboration with Malaria coordinator going so far? R: I think that’s fine.” Midwife coordinator, Sumba  “I: Do you distribute the RDT to each village midwife? R: Yes, I do according their posyandu schedule. We have posyandu schedule every month. I: So do you take care of the distribution only? R: I distribute the RDT and analyse the examination result that was sent by village midwife. So far I got negative result. If there is positive result, I will inform the doctor about that. So the doctor could give medicine that was given to patient through the village midwife. But so far I never get positive result. All result was negative.” Malaria coordinator, Sumba |
|  | 2) Doctors are involved with the treatment of malaria in pregnancy | “I: Just screening? Or you have other roles for prevention? R: No, I just screen so far. For prevention, promotion are done by the midwives in posyandu. Since doctor here is only one. I: Oh yes. What about the treatment? R: What do you mean? I: Treatment for malaria in pregnancy. R: Oh yes, after screening, if the result is positive, midwife would send them to me. I would then treat them.” Doctor, Sumba  “I: So, as a doctor, what role do you play in malaria screening test? R: My role, doctor’s part here is when there are malaria in pregnancy occurs it will be consulted to the doctors, then the doctors will provide therapy to this pregnant woman.” Doctor, Mimika |
|  | 3) Midwife coordinators carry out screening & collect data from various sites | “I: How was the reporting going so far? R: The reporting? I: Yes. R: Malaria report is integrated with KIA (Mother and child health) report. There are some items that should be filled in that report for example we have to put the result of malaria examination at Puskesmas or at village or at Posyandu. On 26th every month, I receive report from all village midwives who have responsibility to make that report. Then I will put their data in the registration book at Puskesmas. I classify the posyandu into each village so we could see what programs that were done at each village.” Midwife coordinator, Sumba  “ I: How do you do your reports of pregnant women to health department? R: For reports, actually each post makes their report to be submitted at 25 each month. When all reporting from all villages are submitted, the midwife coordinator recaps them all before report it to the health department. It’s the midwife coordinator that reports to the health department. I: So from pustu keep making ee… posyandu? R: From posyandu each posyandu will make usually till 25 each month then we’ll submit to midwife coordinator then the midwife coordinator recap it all into puskesmas’ recap then we send it to health department.” Village midwife, Mimika |
|  | 4) Village midwives’ roles vary depending on what malaria related activities are done at the village sites | “I: No… As a midwife, what is your role in malaria prevention for pregnant women? R: Usually, I give suggestion about using mosquito net, and also advise women to clean their house and try to keep it clean.” Village midwife, Mimika  “I: Em………in general, what is your responsibility? What is your responsibility here in Pustu? R.1 : In Pustu…I am more responsible to Maternal and Neonatal. I: To maternal and neonatal? R.1 : Em…Ee… I: Any specific role that you give? R.2: ”in labour” R.1: if it is a labour in night that would certainly become my responsibility. I: Can you tell me about … the implementation of malaria screening test.. in this Pustu? R.1: The malaria screening test… I: Specifically in pregnancy… R.1: In pregnancy… like a mosquito net distribution… and then... if it was her first visit, we would check for malaria.” Village midwife, Mimika |
|  | **Health provider training** |  |
|  | 1) Never been trained specifically on malaria screening | “I: Have you been trained for malaria screening specifically? R: Nothing!” Village midwife, Sumba  “I: Is there ever any information from the hospital or BKIA that a pregnant woman who comes for the first time should do a malaria screening test? R: Never. As far as I know of, only puskesmas that has ever do training on malaria during pregnancy. We have never done it.” Midwife coordinator, Sumba  “I: Have you ever being trained for screening test implementation? R: I have not trained for malaria screening test. I: What about a screening test in pregnancy? R: Not yet. I: Have not been trained, yes? R: I have not.” Village midwife, Sumba  “I: Have you ever get the training to implement this type of single screening and treatment? R: no, not yet. I: not yet… how about the ANC process in IHC? R: IHC for pregnant women? I: yes, the ANC process? R: At least I know what to for malaria treatment, but I don’t know the process in detail. Usually for IHC screening, it is just for general pregnant women screening.” Village midwife, Mimika  “I: I see.. have you ever join any malaria screening test training? R: No, I have not. I: How about the other midwives? R: They have not either. I: So there has never been any counselling from puskesmas? Belum ada sosialisasi dari puskesmas? R: No, never for malaria screening test. I: for malaria in pregnancy screening test? R: Never.” Village midwife, Mimika |
|  | 2) Received training on malaria screening | “I: How is informed to all village midwives that all pregnant women have to be malaria checked? R: Through meeting or training. I: Has the training been done? R: There has been training from malaria program, but I don’t exactly the materials. I: Have they been trained to do it? R: I don’t really remember it! But, as I know, there has been training about malaria screening in pregnancy.” Midwife coordinator, Sumba  “I: Have you ever got training about the single screening and treatment program? How about you Ms. N? R.2: Yes, I have I: When? R.2: It was in 2012.. I: Where was the training held? R.2: At Jayapura. I: What did you get from that training? R.2: The training talked about this (The participant points to the ANC and Malaria screening Poster) Kina is used for first trimester of pregnancy and for the second and third trimester of pregnancy we use OAM. I: It means that program has been implemented since 2012? R.2: Already.” Lab technician, Mimika |
|  | 3) Not received formal training but learned from colleagues | “I: Is there any training about RDT examination? R: Mm.. I: In here? R: Oo, there is no special training for that. We learn it from the other colleagues. We can also ask to laboratory staff about the new information relating to RDT. So we do not always need training to learn something we can learn it by ourselves. If there is staff joins in the training, usually that staff will share the information she/he got from training so that the others staff could be know. That’s what happened in here.” Midwife coordinator, Mimika  “I: How about the training to use RDT? R: We never got the training to use RDT because all of us already knew how to use that from our colleague. I: From your colleague? R: Yes. There is no training about that.” Lab technician, Sumba  “I: Have you ever being trained for RDT? R: I have never. But I’ve seen how to use it. I: Where did you see how to use RDT? R: When we were at MBS, it was from Eijkman. Before Eijkman we had MBS from the province. I: Did they train you or…? R: It was not a training we just looked at how to use it. I: Did they ask you to come with them to see how they do it? R: Yes. But before that in Puskesmas, before me there was this lab technicians who used to worked here, but now that person is assigned at Pustu. S/he used to teach me how to use these equipments.” Lab technician, Sumba |
|  | HMIS | *No quotes* |
|  | **Sustainability of funding** |  |
|  | 1) Global Fund may have stopped funding SST related logistics | “I: This malaria screening test is helped and being funded by global fund, how is the district ability to continue this malaria screening process? R: Dilemma that is happening in district autonomy, that the funding in the district government services is not only for the health sector, there are other sectors also need to be funded. Meanwhile the health sector does require a lot of funding. That is why, we are so grateful there are NGOs who always helps. For the question, global fund is no longer helping, so what the government need to do is to adopt or continue like the district government ability to help. Don’t stop is the key, but it is totally depend on the district government’s political will. The technical worker needs to give good argument to the district government so this can be a top priority in the APBD.” DHO, Sumba  “I: Right. About malaria program funding. Could you please tell me where is the funding from, is it by the headquarters or is it from the local budgeting? R: Until 2012, Global Fund still funded us for malaria, but then it disappeared slowly and then it’s gone. In government sector, it’s quite complicated, probably because our advocacy isn’t good enough, this results in malaria budgeting for the last year wasn’t implemented ass it was planned in 2014.” DHO, Mimika |
|  | 2) No specific budget for malaria in pregnancy | “I: May I know about the budget? I mean is there any special budget for Malaria prevention for pregnant woman program at Puskesmas? R: Malaria prevention for pregnant woman… I don’t think that Puskesmas has budget for that.” Head of Puskesmas, Mimika  “I: Ee.. Then ee.. How do you make the budget arrangement? R: Haah..? I: The finance, the budget? R: What budget? I: The budget that allocated for malaria case management especially malaria in pregnancy? R: We don’t have budget for that. I: No budget for that? R: There is no budget for that. We provide the health service when we go to the field.” Head of puskesmas, Mimika |
